# Vegans, Vegetarians, and Omnivores: How Does Dietary Choice Influence Iodine Intake? A Systematic Review

**DOI:** 10.3390/nu12061606

**Published:** 2020-05-29

**Authors:** Elizabeth R. Eveleigh, Lisa J. Coneyworth, Amanda Avery, Simon J. M. Welham

**Affiliations:** Division of Food, Nutrition & Dietetics, School of Biosciences, The University of Nottingham, Sutton Bonington LE12 5RD, UK; elizabeth.eveleigh@nottingham.ac.uk (E.R.E.); lisa.coneyworth@nottingham.ac.uk (L.J.C.); amanda.avery@nottingham.ac.uk (A.A.)

**Keywords:** iodine status, iodine intake, iodine deficiency, vegan, vegetarian

## Abstract

Vegan and vegetarian diets are becoming increasingly popular. Dietary restrictions may increase the risk of iodine deficiency. This systematic review aims to assess iodine intake and status in adults following a vegan or vegetarian diet in industrialised countries. A systematic review and quality assessment were conducted in the period May 2019–April 2020 according to Preferred Reporting Items for Systematic Reviews and Meta-Analyses (PRISMA) guidelines. Studies were identified in Ovid MEDLINE, Embase, Web of Science, PubMed, Scopus, and secondary sources. Fifteen articles met inclusion criteria. Participants included 127,094 adults (aged ≥ 18 years). Vegan groups presented the lowest median urinary iodine concentrations, followed by vegetarians, and did not achieve optimal status. The highest iodine intakes were recorded in female vegans (1448.0 ± 3879.0 µg day^−1^) and the lowest in vegetarians (15.6 ± 21.0 µg day^−1^). Omnivores recorded the greatest intake in 83% of studies. Seaweed contributed largely to diets of vegans with excessive iodine intake. Vegans appear to have increased risk of low iodine status, deficiency and inadequate intake compared with adults following less restrictive diets. Adults following vegan and vegetarian diets living in countries with a high prevalence of deficiency may be more vulnerable. Therefore, further monitoring of iodine status in industrialised countries and research into improving the iodine intake and status of adults following vegan and vegetarian diets is required.

## 1. Introduction

Vegan and vegetarian diets have gained popularity over the past decade. Characteristically, vegans do not consume any animal-derived products including eggs, dairy, meat, and fish. Vegetarians exclude meat and fish but may consume milk and eggs. A subclass of the vegetarian diet may consume fish but not meat, termed pescatarians. Despite these definitions, varying levels of strictness and adherence to dietary restriction exist at the level of the individual [1]. 

The prevalence of vegetarian and vegan diets differs globally. In developing regions, meat-free diets are traditionally adopted owing to religious, social, ecological, or economic constraints as opposed to personal choice [2]. In industrialised countries, most individuals are afforded the choice of food consumption and level of dietary restriction. Populations in developed countries may adopt these diets for environmental, ethical, religious, health beliefs or social reasons. Presently, well-planned vegan and vegetarian diets have been regarded by the British Dietetic Association and other organizations in industrialised countries to be suitable throughout the lifespan, inclusive of infancy and pregnancy [3,4,5]. However, concerns have been raised regarding the ability of these diets to adequately provide essential micronutrients, such as iodine [6].

Iodine is an essential micronutrient, required in trace quantities, which is vital for the synthesis of thyroid hormones—triiodothyronine (T3) and thyroxine (T4) [7]. The thyroid hormones are crucial for the regulation of metabolism, growth, and neurological development [8]. Iodine deficiency presents as a spectrum of clinical disorders termed ‘iodine deficiency disorders’ (IDD’s) which occur when recommended intakes are not achieved (150 µg day^−1^) [8]. These include hypothyroidism, goitre abnormal thyroid nodular pathology, and cretininism in infants born to mothers with a low iodine status during pregnancy and lactation [7,9]. Low iodine intake may be a risk factor for thyroid nodule formation, particularly in females [10,11]. Most nodules are harmless; however, some may result in thyroid dysfunction or malignancy [11]. Excessive iodine intake (>1000 µg day^−1^) may lead to hyperthyroidism in individuals with preexisting thyroid disease or iodine deficiency [12]. Iodine deficiency is not limited to developing countries—mild–moderate deficiency exists in industrialised nations including Europe, UK, Australia and select populations in the USA [9]. In 2011, iodine nutrition was highlighted as a significant public health concern following estimates indicating that 2 billion people globally were deficient [13]. Recent data collected by the WHO show a global decline in iodine deficiency between 1993 and 2019, suggesting that less than 8.5% of the world’s population are affected [14]. However, subgroups of European populations are still at increased risk of iodine deficiency [6]. 

Iodine deficiency traditionally was assessed by monitoring the prevalence of visible goitre in populations [7]. After the development of newer methods for measuring iodine status, it was recognized that low-level deficiency may be present in industrialised populations not displaying obvious thyroid enlargement [15]. Various biomarkers can be used to estimate population iodine status and intake [15]. Urinary iodine concentration (UIC) is the most common and practical marker [16]. This is because >90% of the iodine ingested from dietary sources is readily excreted in the urine [17]. Spot UIC and 24 h measures can be used to detect and monitor iodine adequacy and deficiency. However, these estimates only correspond to recent intake [16]. Additionally, thyroid function tests are required routinely to detect iodine adequacy in vulnerable populations such as pregnant and/or lactating women and infants [16]. Dietary iodine can be estimated indirectly by UIC or by common dietary assessment methods [15]. Limitations of the methods used must be considered. Biomarkers of status and dietary intake methods are not always the same between studies which adds to the challenge of reliably comparing iodine amid populations [18].

Individuals residing in developing countries, who are reliant on plant-based foods in their diet, have a higher prevalence of iodine deficiency [19]. The bioavailability of iodine from plant sources has been suggested to be determined by rainfall and water collection on crop leaves with much of the iodine within plants not being bioavailable [20]. In industrialised countries where people consume a ‘Western diet’, the key dietary sources of iodine are bread fortified by iodised salt, cow′s milk, and dairy products [21]. Seafood, eggs, and seaweed are also iodine rich but are not regularly consumed [22]. Water and salt iodination strategies are present in most states in the US and select countries in Europe [23,24]. Countries such as the UK have yet to establish a mandatory salt fortification program and despite regular manufacturing of iodised salt, it is not widely available for public purchase [25]. 

For this reason, individuals who consume diets excluding iodine-rich food, principally dairy, eggs, and/or fish, have increased risk of iodine deficiency [26]. Further complicating this issue is the growing availability and acceptance of plant-based food ‘alternatives’, regularly consumed by vegans and vegetarians, that naturally have negligible iodine content and are not regularly fortified [27,28]. The size of the plant-based ‘alternatives’ food market has been reported to have almost doubled between 2014 and 2017 in the UK [29]. 

Currently, two reviews exist investigating iodine in the diets of vegans and vegetarians, one in 2005, which was updated in 2009 by the same authors [30,31]. The most recent review included eight studies, covering a period between 1981 and 2003, with the conclusion that strict vegans and vegetarians living in Europe have iodine values below recommended levels and are at risk of deficiency. In the years since publication, these diets have become more widely accepted and it is likely that food consumption practices have changed considerably since this last assessment of iodine intake in adults following vegan and vegetarian diets. Given the potential health consequences of iodine deficiency, it is important to re-examine whether adults following either a vegan or vegetarian diet are still at risk of iodine deficiency.

Thus, the aim of this review is to assess the iodine intake and status in adults following a vegan or vegetarian diet in industrialised countries across time. The objectives included (1) evaluation of the methods used to assess iodine; (2) determination of the iodine intake and food consumption in vegan and vegetarian adults; (3) assessment of the iodine status and prevalence of iodine deficiency using urinary iodine concentration (UIC); (4) comparison of the iodine intake, status and prevalence of deficiency between vegans, vegetarians and omnivores; and (5) consideration of gender differences in estimates of iodine nutrition.

## 2. Materials and Methods 

This systematic review was according to the Preferred Reporting Items for Systematic Reviews and Meta-Analyses (PRISMA) checklist [32]. 

A systematic search of literature was performed from 20 May 2019 to April 2020. Electronic databases (Ovid MEDLINE, Embase, Web of Science, PubMed, and Scopus) were searched using text terms with appropriate truncation, and Medical Subject Headings. Search term sensitivity and relevance of article identification was tested using preliminary searches in Ovid MEDLINE (Supplementary Table S1). All database searches were refined by ‘Humans, Adults (aged < 18 years) and English Language’. Identified relevant studies were saved onto EndNoteTM online and duplicates were removed. To limit bias, relevance was confirmed by two investigators. Additional relevant articles were sourced from reference lists of included studies. 

The current systematic review addressed study eligibility using the population–intervention–comparison–outcome (PICOS) formulation (Table 1) [33]. Additionally, only articles with full paper availability published in/after 1990 were considered for inclusion. 

Data extraction was completed independently. The terms used for data extraction were discussed and finalised by two secondary researchers. A modified version of “Data collection form for intervention review—RCTs and non-RCTs” by The Cochrane Collaboration was used for data extraction [34]. Adaptions considered the characteristics of interest and study design. To permit comparison between groups, ‘moderate vegans’ were considered as vegetarians. ‘Mixed diet’ and ‘meat eaters’ as omnivores and ‘living food dieters’ as vegans. Due to variation in nomenclature, demi vegetarians will be considered separately. To make comparisons between genders, where possible, data on males and females were extracted separately. 

Following data extraction, study quality was critically appraised by one author. Quality was assessed using the National Heart, Lung, and Blood Institute (NHLBI) Quality Assessment Tool for Observational Cohort and Cross-Sectional Studies [35].

According to Guidance for Assessing the Quality of Observational Cohort and Cross-Sectional Studies provided by NHLBI, fixed response was selected for three questions to account for the nature of cross-sectional studies. Exposures and outcomes are measured and assessed during the same timeframe excluding time to see an effect and often lack a follow up, hence questions 6 and 7 would automatically receive a “NO” response. Additionally, question 13 was given a fixed response of “NA” (Supplementary Table S2). Quality of matched-pair interventions was assessed using the Quality Assessment of Controlled Intervention Studies, and Matched-Pairs (Case-Control) by The Quality Assessment of Case-Control Studies NHLBI [35]. Assessment was completed by one author and reviewed by another independent assessor prior to agreement (Table 2).

The WHO criteria for assessing the severity of IDD (1994) stratified by median urinary iodine concentration (UIC) was used to assess the relative level of deficiency in each dietary group. According to this classification, the rate of deficiency is described as the percentage of individuals in each group with UIC below <100 or <50 µg L^−1^, in severe deficiency [6]. 

Funnel plots were generated for both UIC and dietary iodine intake data. For urinary iodine status, summary values and *number of participants* for each dietary group (Table 3) were used to generate an overall *population mean* value (*µ*).

$$Population\;mean\;\left(\mu \right)=\frac{\sum \mathrm{UIC}\;values}{total\;number\;of\;participants}$$

The standard error for each observation group was generated according to the equation:

$$SE=SQRT{\left(SQRT{\left(\mu \times \frac{1-\mu }{number\;of\;subjects}\right)}^{2}\right)}^{}$$

Confidence limits were generated as indicated below.

95% CI=μ±(1.96×SE)99.7% CI=μ±(3×SE)

Confidence limits were generated for each population studied and used to generate funnel plots of UIC or iodine intake shown against study size (Supplementary Figures S1 and S2).

## 3. Results

**Figure 1:**
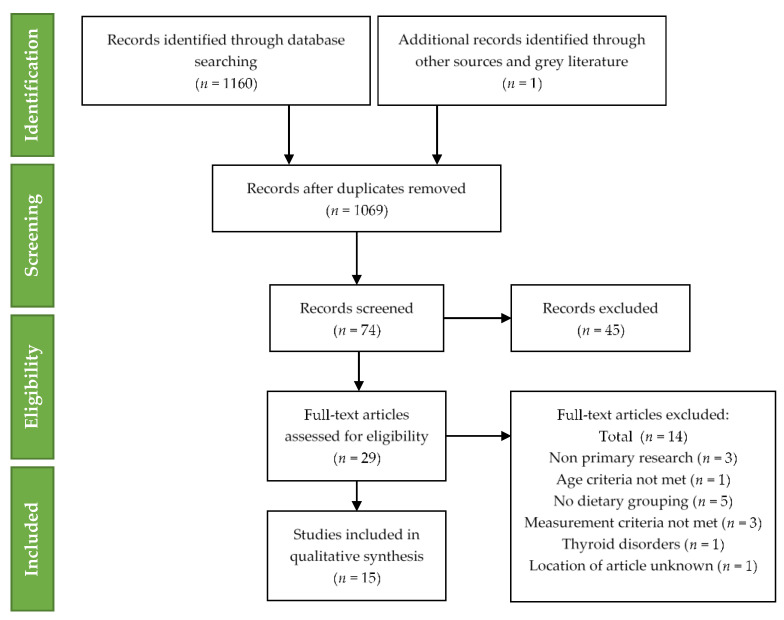
Preferred Reporting Items for Systematic Reviews and Meta-Analyses (PRISMA) flow diagram for included studies.

The following exclusion of studies qualitative synthesis was completed for fifteen studies. The technique of study selection along with the number of included and excluded studies recorded for this systematic review is shown in the PRISMA 2009 flow diagram (Figure 1) [32].

Fifteen relevant studies were identified examining the iodine intake or status by dietary group Table 4. Consistent with scientific literature, different descriptors and nomenclature were used to define vegetarian diet types (Supplementary Table S3). Three studies used objective assessments to group individuals [43,45,46].

### 3.1. Urinary Iodine Status

**Figure 2:**
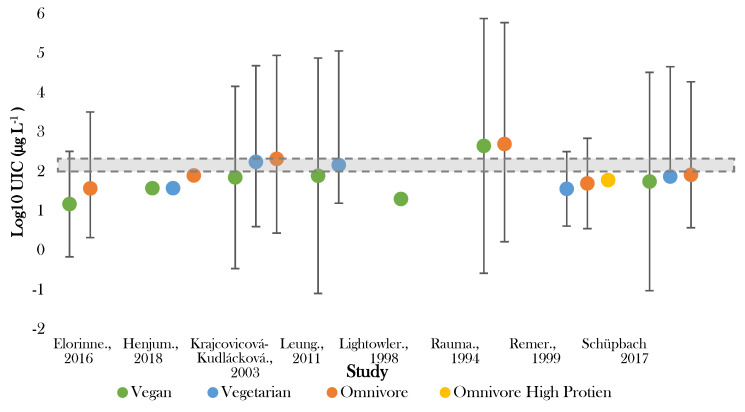
Visual representation of iodine status by median urinary iodine concentration (UIC) for included studies. The shaded grey bar represents the optimal range for iodine status (100–299 μg L^−1^). Significance values are not presented within this figure. See Table 3.

Eight studies investigated iodine status by urinary iodine concentration (UIC) (Table 3; Figure 2). Four studies measured UIC using spot samples [38,40,44,48]—of which, one study collected multiple fasted samples to determine average values [44].

The lowest median UIC (16.8 μg L^−1^) was recorded by Lightowler in UK male adults following vegan diets [41]. Rauma [50] reported the highest median UIC (<500.0 µg L^−1^) in Finish omnivores. Large variation in UIC existed in several studies [40,44,50], with one study showing variation in those following vegan diets between <200 and 1700 µg L^−1^ [50]. The majority (75%) of the recorded values for UIC fell below the expected population mean of 95.6 µg L^−1^ (Supplementary Figure S1) and half of the values fell either on or outside of the 99.7% confidence limit. 

In all studies giving intergroup comparisons, the lowest median UIC was recorded for those following vegan diets and the highest for omnivores [38,39,40,44,47,48,50]. Five out of eight studies recorded median UIC in vegans to be significantly lower than omnivores (*p* < 0.005) [38,39,40,44,47]. All studies observed UIC in those following vegetarian diets to be higher than vegan diets, yet lower than omnivorous diets [38,39,40,44,47]. The difference between vegetarian and omnivorous diets was significant in three studies (*p* < 0.05) [38,39,47]. 

IDD assessment according to the WHO criteria ranged from severe (inadequate) to at ‘risk’ of adverse health consequences (excess) across studies [51]. Supplementary Table S4 presents national data corresponding to countries of included studies.

Optimal status (100–200 µg L^−1^) was achieved in vegetarian groups in Slovakia and Boston [39,40]. No adults following vegan diet had median UIC within the optimal range [38,39,40,41,44,48,50]. Seven studies observed one or more dietary group below the cut off for optimal population UIC [38,39,40,42,44,47,48]. Iodine deficiency (mild–severe) (50–99 μg L^−1^–>20 μg L^−1^) was recorded in adults following vegan diets in six studies [38,39,40,42,44,48], vegetarian diets in two studies [44,47], and omnivorous diets in four studies [38,44,47,48]. 

Those following vegan diets were most frequently seen to exhibit either mild (50–99 µg L^−1^) or moderate deficiency (20–49 µg L^−1^) [12,38,39,41,44,48], and in two studies were found to be severely deficient (<20 µg L^−1^) [40,47]. In one of these studies, >75% of those following vegan diets fell into the severely deficient category [48]. Both those following vegan and vegetarian diets were more commonly observed to be moderately deficient, whilst, conversely, omnivores were found in two studies to exhibit excessive iodine status [39,50]. This was also noted for those following vegan diets in one Finnish study [50].

### 3.2. Dietary Iodine Intake 

**Figure 3:**
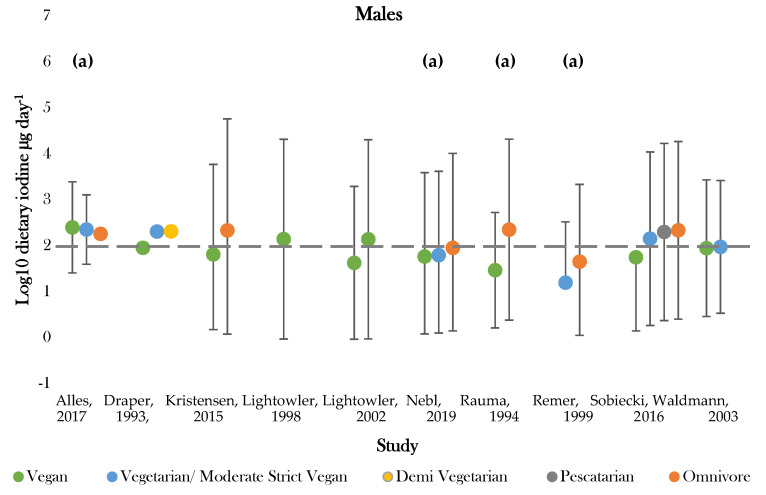

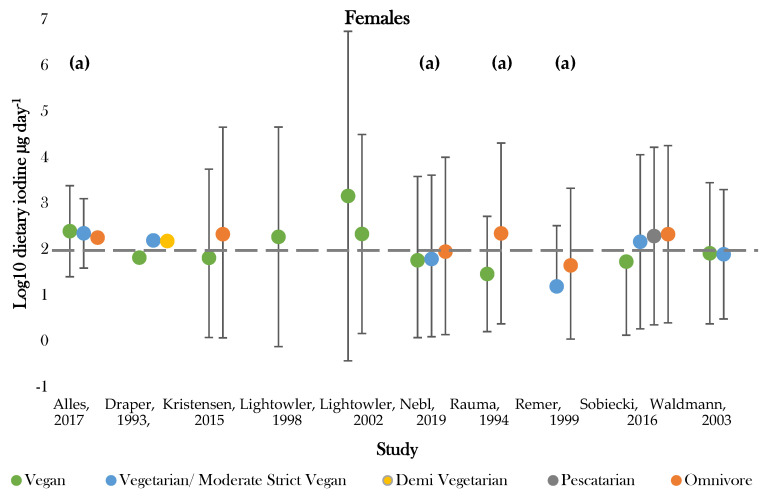
Visual representation of estimated average iodine intake (µg day^−1^) for included studies. The grey dashed line represents the adequate intake recommended by the WHO of 150 µg day^−1^. (**a**), mixed gender values. Significance values are not presented within this figure. See Table 5.

Methods for assessing dietary intake are listed in Supplementary Table S5. Ten studies reported estimates for daily iodine intake [36,37,41,42,43,45,46,47,49,50]—of which, four studies were investigating iodine specifically [41,42,47,50]. The additional studies investigated other macro- and micronutrient intakes besides iodine (Table 5; Figure 3) [36,37,43,45,46,49].

The highest daily iodine intake was recorded in females following vegan diets of 1448.0 ± 3879.0; 29.0 ± 18.0 µg day^−1^ [42,50]. The lowest dietary intake was found in those following vegetarian diets of 15.6 ± 21.0 µg day^−1^ [47]. Seven studies assessed iodine intake between vegans and one or more dietary group [36,37,43,45,46,49,50]. Omnivores (male and female) had the highest estimated average intake in 85% of studies [43,45,46,47,49,50]. Vegan groups tended to have the lowest iodine intake. Males following vegan diets had average intakes lower than all comparative dietary groups in all studies, apart from that conducted by Allès (2017) [36]. Females following vegan diets presented the lowest iodine intake in 75% of studies [37,45,49]. Varied intakes were recorded for moderate vegans, vegetarians, and pescatarians across studies, with estimates ranging between 222.6 ± 1.1 and 15.6 ± 21.0 µg day^−1^ [36,43,47]. The majority of values fell around the expected population mean (184.1 µg day^−1^), although there was a general tendency for values to be slightly below this level (Supplementary Figure S2).

Recommended criteria for iodine intake varied according to country of study. Comparisons were, therefore, drawn according to recommended intake values denoted by the WHO, with values above 150 µg day^−1^ being classed as adequate [51]. One study recorded estimates above the adequate range for all dietary groups [36]. Omnivores most frequently achieved adequate intake, with only two studies recording intakes below 150 µg day^−1^ for both genders [43,47]. Those following vegan diets most frequently showed dietary iodine inadequacy. Adequate intake was recorded for 44% of female and 66% of mixed gender estimates [36,37,41,42,43,45,46,49,50]. No groups of males only had intakes above the adequate cut off values [37,41,42,49,50]. Intakes for moderate vegans (vegetarians), vegetarians, and pescatarians were similar between genders and were above adequate in half of studies [36,37,43].

Only four studies investigated the relative consumption of different food groups [36,37,43,46]. None reported the actual contribution of each food group to dietary iodine independent of iodised salt and supplements. However, adults following vegan and vegetarian diets tended to report significantly higher consumption of plant-based food groups (fruit, vegetables, legumes, tubers cereals and grains) [43,46], along with tofu and soya-based products which naturally have a low iodine content. One study reported the consumption of milk alternatives in each dietary group, and vegans had the largest consumption of plant-based milk alternatives [36]. As expected, milk and dairy, egg, and fish consumption was significantly higher (*p* < 0.0001) in vegetarians and omnivores compared to vegans [36,43].

Six studies measured seaweed consumption [37,41,42,45,49,50]. Adults following vegan diets had the greatest seaweed intake, with four studies stating that consumption significantly increased dietary intake such that iodine intake would be considered ‘excessive’ [37,41,42,49]. Seaweed was not regularly consumed by omnivores and moderate vegans (vegetarians), vegetarians, demi vegetarians or pescatarians [37,45,46,47,49,50].

Mandatory salt fortification programs were present in three countries of included studies (Table 6) [39,40,49]. Eight studies did not record the contribution of iodised salt to dietary iodine [36,37,41,42,43,45,46,49].

Supplement intake was recorded in seven studies [37,41,42,43,45,46,49]. Two studies did not record iodine-specific supplementation [45,46]. One study prevented supplement intake during the study and another excluded supplement contribution in dietary analysis [42,47].

Three studies recorded supplement intake in adults following vegan diets, contributing between 9.0 and 54.0 µg day^−1^ to dietary intake [37,41,49]. Supplements contributed greater iodine to diets of omnivores (78.9 µg day^−1^–107.0 µg day^−1^) [37,49]. No supplement consumption was recorded for moderate vegans (vegetarians), vegetarians, demi vegetarians or pescatarians [37,43,45,46,47].

## 4. Discussion

The popularity of vegan and vegetarian diets has increased considerably in the past decade. The prospect of these diets acting as a barrier to adequate iodine nutrition could increase the risk of developing preventable health consequences associated with deficiency and furthering the worldwide public health issue of iodine [60]. Given that these diets are commonly followed by females of childbearing age (16–24 years), lowered dietary iodine intake in these groups could significantly impact future generations and reduce societal productivity [6,61,62].

The present systematic review is the most recent review to evaluate the evidence for iodine intake and status among adults following vegan and vegetarian diets [30]. Our review showed that the discourse has not changed and that adults following a vegan diet, living in industrialised countries, not consuming seaweed or iodine-containing supplements, appear to have increased risk of low iodine status, iodine deficiency and inadequate iodine intake compared to less restrictive dietary groups. Comparison of dietary estimates supports the possibility of a gender difference within vegan groups, as no vegan males achieved adequate intake. Vegetarians or pescatarians are more at risk of low iodine status and intake compared to omnivores but not vegans.

These results are in accordance with the previous review by Fields, Dourson and Borak in 2009 [30], whereby it was concluded that iodine adequacy decreased with increased dietary restriction [30]. However, the previous review did not highlight that some study populations following omnivorous diets also had low iodine status and mild–moderate deficiency [30]. The degree of vulnerability in all dietary groups appears to be impacted by not only individual dietary choices, practices, and restrictions, but country-specific dietary determinants and national food fortification strategies. For this reason, vegans and vegetarians living in industrialised regions where national population iodine measures are below adequate or where dietary intake is insufficient are more susceptible to iodine deficiency. This trend can be observed by comparing median UIC (MUIC) of included studies with the corresponding national data from the country of origin. MUIC tends to closely represent that of omnivorous diets, owing to these practices being the dominant in most industrialised countries.

Vegan and vegetarian MUIC in all studies, apart from that conducted by Rauma (1994), fell below national values [50]. In half of studies, the extent was substantial enough for adults following vegan and vegetarian diets to be classified in a lower deficiency category, according to the WHO criteria, than omnivores and national data—for example, mild (omnivores)–moderate (Vegans and vegetarians) [38,39,40,48]. This trend can be explained by exploring values determined by Henjum (2018) of non-pregnant young women in Norway [38]. Omnivores in this study presented UIC values concordant with current Norwegian national data (80.0, 75.0 µg day^−1^), which could be due to efficient data collection of northern, western and eastern regions of Norway [14,38]. As omnivorous diets are the most dominant in industrialised countries, this sample is likely to be representative of the Norwegian population. Omnivorous MUIC and national data are indicative of mild deficiency, whereas for those following vegan and vegetarian diets, MUIC is suggestive of moderate deficiency, therefore indicating that the susceptibility of vegan and vegetarian populations to deficiency is in part dependent on national iodine status.

This trend is not apparent in woman of reproductive age in Switzerland, despite lower observed values in those following vegan diets, as all groups and national data would be classified as mildly deficient [44]. This could be explained by regular consumption of iodized salt in Swiss households, which is greater than 80% [63]. Fortified bread is a significant contributor to dietary iodine and can be consumed by all dietary groups [63]. A reduction or restricted legislation in iodine fortification could increase the risk of iodine deficiency. In Germany, Remer (1999) induced deficiency in vegetarian and omnivorous diets when preventing consumption of fortified and iodinated foods under controlled conditions [47]. In this study, daily iodine intake dropped below requirements required for optimal thyroidal function (50.0–80.0 µg day^−1^). Disuse or lack of availability of iodine-fortified foods could produce similar consequences.

Shifting legislation reflects changes in iodine status by dietary group over time in Finland from a risk of excess to moderate–severe deficiency in 2016 [48,50]. This is following a reduction in iodised salt consumption possibly influenced by efforts to reduce salt consumption in the general public and replacement of the traditional diet rich in fish and dairy for diets providing improved fruit and vegetable intake [64]. The Finnish Vegan Society was founded in October 1993 [65], a year before Rauma’s study was conducted, and food consumption is likely to have changed in this time, as these diets have become widely accepted. Papers published prior to 1990 were excluded, as meat-free diets did not appear in the mainstream until the 1980s, and it is likely that food choices and landscape have changed substantially since this period [66]. Recent studies published after 2010 represent almost half of the included studies which could account for the ‘second-wave’ vegetarian movement supported today [12,38,43,44,45,48,49].

Despite the popularity of vegan and vegetarian diets, there is no homogenous term to define these dietary groups, thus questioning the accuracy of group estimates in included studies. Three studies used objective assessments to group individuals by dietary preference [43,45,46]. Self-reported measures are frequently inaccurate at determining dietary preference [30]. Misreporting may additionally be a consequence of misinterpretation of the term “vegetarian” and its associated dietary restrictions or, additionally, over exaggeration of restrictive practices to align with vegetarian ideology [30,67]. Juan, Yamini and Britten (2015) observed errors with adherence to vegetarian diets when investigating food consumption patterns of the U.S. population in the period 2007–2010 [67]. The authors determined that half of those identifying themselves as vegetarian consumed meat, poultry, or seafood and, therefore, would not be described as vegetarian using typical definitions. This issue was discussed previously in the systematic review conducted by Fields, Dourson and Borak, indicating that individual dietary intake based on the description of typical “vegetarian” regimes from self-reports may be inaccurate and indicates that consistent strict dietary adherence in the included studies is likely to be low [30].

The duration of practicing a specific dietary preference must be considered when examining the diets of vegan and vegetarians. Most studies (apart from that conducted in experimental conditions and studies of recreational runners) included participants with 1-year minimum dietary adherence [43,47]. Draper (1993) only recruited participants who had elected to adopt their diets in adulthood and had not grown up as vegetarian [37]. This raises the question as to whether the length of dietary adherence affects iodine measures. Long-term vegetarians could be better at planning their diet adequately if receiving guidance from their parents from a young age or iodine intake could reflect reduced diet diversity and long-term dietary compliance.

Reduced dietary intake for those following vegan diets may reflect difficulties in accurately measuring dietary intake. Intake can be measured using various methods. The “gold standard” for estimating iodine intake is a dietary record or food diary inclusive of one weekend day [17]. Seven included studies followed this methodology, additionally pairing food diaries with weighed or duplicate records [36,37,41,42,43,47,49]. Food diary measurements reflect recent intake; therefore, 10 days are recommended for iodine to account for food items that are rich in iodine but not regularly consumed [68]. Only two studies collected records exceeding this duration [46,50]. The most frequent length of estimates was 4 days, which is acknowledged as an adequate length to record dietary intake [69]. Duplicate dairies are suitable for valuation of minerals such as iodine [70]. This technique is useful for foods with iodine values not contained within food tables or composition data. For this reason, this technique is useful to assess the dietary iodine of those following vegan diets who may consume foods that are novel or not frequently eaten by the general public.

Two studies used validated FFQs to estimate iodine intake [45,46]. FFQs are often designed to be specific to a nutrient of interest and aim to include food items contributing to intake. Both studies had developed FFQs to address intake of multiple micronutrients and were not iodine specific. As FFQs are a comprehensive list and not all foods can be contained, they are, therefore, limited to the listed items. It is unlikely that these studies addressed foods that largely contribute to iodine intake but are not regularly consumed such as seaweed or novel foods consumed by vegans. In addition, FFQs have been recognised to frequently overestimate iodine intake as supported by previous validation studies [71].

For all studies, estimations of iodine intake were calculated using mean iodine concentration of foods and beverages provided by regional food composition tables or databases and are, therefore, country specific [36,37,41,42,43,45,46,47,50]. Estimates based on one summary statistic cannot account for variation in iodine content between food items across time, season, and geographic location—for example, the iodine content of milk, which also varies according to processing and product origin [72]. In vegan adults, the lowest intakes were recorded in Finland (29.0 ± 18.0 µg day^−1^) [50]. Krajcovicová-Kudlácková (2003) discussed that Finish food tables tended to estimate lower iodine intake compared to British food tables [39], and variation in this case probably represents geographical diversity between countries and food availability. Waldmann (2003) [46] created a database accounting for vegan food items regularly consumed in Germany, thus improving estimates. Four studies were conducted in the 1990s [37,41,47,50]. It is likely that food tables and databases have improved availability of the iodine content of foods. However, these methods lack iodine values for newly consumed food and products, particularly those gaining in popularity such as plant-based alternatives.

Adults following vegan diets had the largest consumption of plant-based milk alternatives. Bath et al. investigated the iodine concentration of milk alternatives available in the UK in 2015 and determined the concentration of unfortified milk-alternative drinks (median 7.3 μg kg^−1^) to be significantly lower than the dairy milks analysed (median 438.0 μg kg^−1^) [28]. Those consuming alternative milks may not be aware of the lower contribution of plant-based milks to iodine and need to ensure intake from other sources.

The highest iodine intake was recorded for females following vegan diets, living in London (1448.0 ± 3879.0 µg day^−1^), whose regular consumption of seaweed increased intakes to over six times the RNI [42]. Five studies recorded seaweed consumption in vegan diets only, observing intakes close to, or over, the maximum tolerant level [37,41,42,46,49]. Seaweed is a naturally rich source of iodine, but the relative content is highly variable and can provide excessive quantities and, therefore, regular consumption is not recommended [73]. Seaweed is not customarily consumed in the Western diet. However, it has recently become popular in UK food products as a whole food and functional ingredient [74]. For example, carrageenan is widely used in newly formulated vegan and vegetarian products. Manufacturers use it to replace gelatine, as it is derived from plant origin [74]. Draper (1993) observed low iodine intakes in adults following vegan diets in London despite 95% recording regular consumption of seaweed or foods containing seaweed powder [37]. While the iodine content of seaweeds is often high, it is not a food that is consumed in large quantities, hence the contribution towards dietary intakes is likely to be small and inconstant [75]. Moreover, iodine content significantly differs between seaweed species consumed [73].

Voluntary fortification is present in most countries [59], yet very few manufacturers add fortified salt to food items and iodinated salt is not easily available to consumers. This appears to be true in the study conducted by Rauma (1994), where only one Finish vegan reported consuming iodised salt regularly [50]. Furthermore, Bath et al. (2013) conducted a shelf survey of five major chain supermarkets in the UK and found that iodized salt was only sold in 42% (32 out of 77) of shops and that the iodine content of fortified salt sold was low (11.5 ± 4.2 μg g^−1^) [25]. Draper (1993) noted the consumption of ‘sea salt’ in 95% of UK adults following vegan diets [37]. Although ‘sea salt’ and other ‘specialty’ salts such as Himalayan sea salt contain iodine, the concentration is relatively small compared to iodised salts [76]. Additionally, of two studies reporting iodised salt intake [37,50], neither identified having adjusted intakes to account for volatile iodine losses experienced during cooking [77]. Kristensen (2015) attempted to quantify the involvement of iodised salt by measuring reported sodium intake [49]. Despite, the routine uses of iodised salt in Danish households, 55 out of 70 vegans failed to meet dietary recommendations for iodine [49]. Low dietary intake in vegans in this study may be explained by reduced dietary sodium intake reported by vegan participants and infrequent consumption of salt-rich processed food items. Moreover, vegan ready meals are not always readily available from retailers.

Limitations of the study. In addition to the limitations highlighted above, it should be noted that the values recorded in the included studies tended to be skewed below the mean estimated population levels for UIC (Supplementary Figure S1) and intake (Supplementary Figure S2). This would suggest that for most studies, the sample population was not wholly reflective of the general population. This is a challenge for such studies, as the cohort of individuals prepared to participate is often characterised by those with an interest either in their dietary choice or dietary control for health reasons. Accessing the wider public is much more difficult and a challenge that needs to be addressed in future studies. There was some overlap with the previous systematic review, and we found that recent studies investigating iodine nutrition were relatively scarce. Further, it was necessary to combine groups, thereby oversimplifying dietary practice to enable comparison of studies. Lastly, there was a lack of values for contribution of specific food groups (e.g., supplements) and iodised salt.

## 5. Conclusions

Iodine deficiency remains a public health problem worldwide and is of concern following the “re-emergence” of iodine deficiency, especially in industrialised countries [78]. This review agrees with findings from the previous systematic reviews exploring this topic [30,31], confirming that vegans and vegetarians, living in industrialised countries, not consuming seaweed or iodine-containing supplements, appear to have increased risk of low iodine status, iodine deficiency and inadequate iodine intake compared to adults following less restrictive diets. The evidence suggests that the degree of vulnerability appears to be relative to the prevalence of deficiency at the national level. This review also highlights the variety of methodological issues associated with estimating iodine in unique dietary groups. In conclusion, further monitoring of iodine status in industrialised countries and research into improving the intake and status of vegan and vegetarian diets is required. Efforts need to be made to devise a means of safe iodine consumption whether by fortification of staple foods or iodised salt provision, in addition to information delivery intended for tolerable consumption of seaweed varieties. Lastly, nutrients frequently lacking in vegan and vegetarian diets should be routinely labelled on foods regularly consumed by these groups in order to highlight to those making these purchases that there is a need to consider the levels that are being consumed.

## Figures and Tables

**Table 1:** Population–intervention–comparison–outcome (PICOS) criteria for study inclusion and exclusion.

Criteria Category	Inclusion	Exclusion
Population	Adults (aged ≥ 18 y) residing in industrialised nations.	Individuals (aged < 18 y), unless results display separate data; adults residing in developing countries; populations with a high prevalence of thyroid disorders.
Intervention/exposure	Participants with any type of dietary preference or restriction. Voluntary or otherwise.	Use of a dietary grouping without defining diet characteristics.
Comparators	Differing dietary preference or restriction.	None.
Outcome measure	Iodine intake or status measured by UIC or analysis of dietary records.	No analysis of iodine intake or status; use of thyroid measures alone for iodine intake and status.
Study design	Any study design with relevant outcomes.	None.

**Table 2:** NHLBI tool for quality assessment of included studies.

	Question														
Study, Year	1	2	3	4	5	6	7	8	9	10	11	12	13	14	Rating
**Observational Cohort Cross-Sectional Studies**															
Alles, 2017 [36]	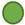 +	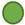 +	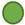 +	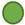 +	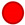 −	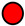 **−**	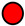 **−**	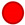 −	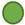 +	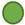 +	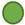 +	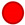 −	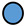 **a**	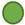 +	Fair
Draper, 1993 [37]	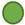 +	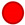 −	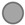 r	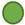 +	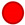 −	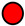 **−**	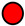 **−**	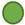 +	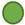 +	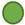 +	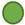 +	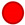 −	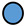 **a**	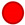 −	Fair
Henjum, 2018 [38]	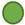 +	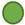 +	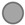 r	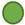 +	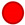 −	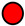 **−**	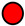 **−**	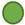 +	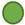 +	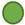 +	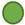 +	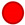 −	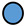 **a**	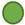 +	Good
Krajcovicová-Kudlácková, 2003 [39]	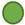 +	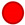 −	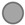 r	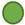 +	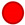 −	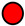 **−**	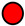 **−**	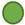 +	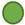 +	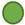 +	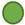 +	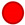 −	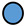 **a**	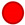 −	Fair
Leung, 2011 [40]	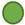 +	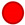 −	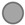 r	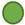 +	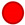 −	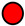 **−**	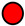 **−**	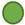 +	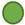 +	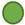 +	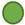 +	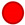 −	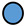 **a**	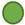 +	Good
Lightowler, 1998 [41]	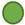 +	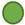 +	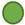 +	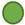 +	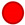 −	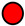 **−**	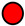 **−**	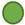 +	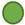 +	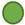 +	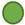 +	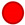 −	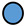 **a**	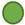 +	Fair
Lightowler, 2002 [42]	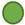 +	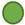 +	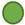 +	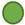 +	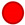 −	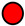 **−**	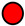 **−**	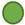 +	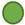 +	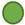 +	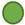 +	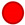 −	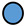 **a**	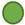 +	Good
Nebl, 2019 [43]	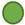 +	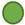 +	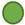 +	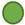 +	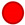 −	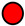 **−**	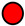 **−**	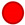 −	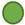 +	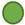 +	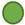 +	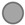 r	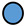 a	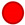 −	Good
Schűpbach, 2017 [44]	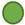 +	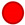 −	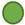 +	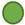 +	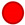 −	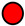 **−**	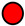 **−**	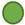 +	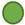 +	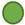 +	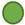 +	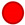 −	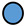 **a**	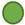 +	Good
Sobiecki, 2016 [45]	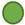 +	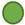 +	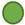 +	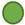 +	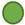 +	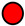 **−**	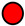 **−**	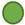 +	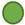 +	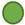 +	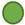 +	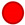 −	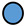 **a**	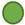 +	Good
Waldmann, 2003 [46]	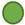 +	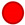 −	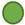 +	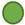 +	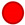 −	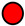 **−**	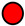 **−**	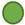 +	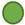 +	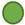 +	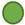 +	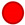 −	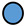 **a**	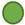 +	Good
**Controlled Intervention Studies**															
Remer, 1999 [47]	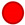 −	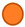 ?	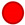 −	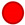 -	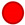 −	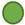 +	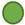 +	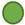 +	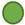 +	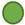 +	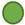 +	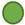 +	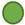 +	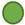 +	Good
**Case-Control Studies**															
Elorinne, 2016 [48]	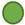 +	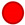 −	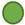 +	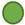 +	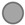 r	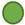 +	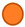 ?	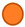 ?	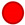 −	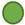 +	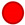 −	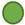 +	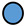 **a**	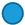 a	Fair
Kristensen, 2015 [49]	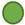 +	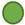 +	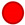 −	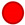 −	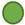 +	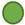 +	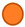 ?	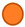 ?	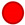 −	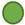 +	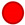 −	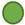 +	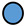 **a**	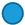 a	Fair
Rauma, 1994 [50]	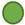 +	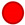 −	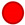 −	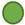 +	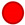 −	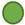 +	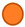 ?	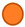 ?	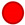 −	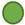 +	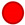 −	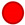 −	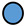 **a**	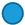 a	Poor

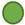
+, yes; 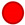
−, no; 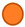
?, cannot determine; 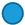
a, not applicable; 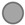
r, not reported; 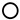
(outlined), fixed answers according to NHLBI recommendations.

**Table 3:** Iodine status and deficiency in vegans, vegetarians, and omnivores in industrialised countries.

Study, Year	Assessment Method	Dietary Group (n) (Male, Female)	Iodine Status by UIC (µg day^−1^)	Criteria for Iodine Deficiency Disorders
Elorinne, 2016 [48]	Spot UIC Sandell–Kolthoff method.	Vegan (21)	15.0 (4.6, 21.8) ^1,^**	Severe
		Omnivore (18)	37.4 (17.7, 86.5) ^1^	Moderate
Henjum, 2018 [38]	Spot UIC.	Vegan (9)	38.0 ^1,^**	Moderate
		Vegetarian (27)		
		Omnivore (367)	80.0 ^1^	Mild
Krajcovicová-Kudlácková, 2003 [39]	24 h UIC Sandell–Kolthoff method.	Vegan (15) (6,9)	71.0 (9.0–204.0) ^2,^**	Mild
		Vegetarian (31) (12,19)	177.0 (44.0–273.0) ^2,^**	Optimal
		Omnivore (Mixed Diet) (35) (15,20)	210.0 (76.0–423.0) ^2^	Optimal with risk of health consequences
Leung, 2011 [40]	Spot UIC spectrophotometry.	Vegan (62) (19,43)	78.5 (6.8–964.7) ^2,^*	Mild
		Vegetarian (78) (26,52)	147.0 (9.3–778.6) ^2^	Optimal
Lightowler, 1998 [41]	Four 24 h UIC Sandell–Kolthoff method reaction.	Vegan (30) (11,19)	Total, 20.2 ^1^, M, 16.8 ^1^, F, 20.5 ^1^	Severe-Moderate
Rauma, 1994 [50]	24 h UIC.	Vegan (Living Food Diet) (10)	<450.0 (<200.0–1700.0) ^2^	Optimal with risk of health consequences
		Omnivore (12)	<500.0 (300.0−1200.0) ^2^	
Remer, 1999 [47]	Two 24 h UIC.	Vegetarian (6) (3,3)	36.6 ± 8.8 ^3,^*	Moderate
		Omnivore (6) (3,3)	50.2 ± 14.0 ^3^	Mild
		Omnivore (High Protein) (6) (3,3)	61.0 ± 8 ^3^	Mild
Schüpbach, 2017 [44]	Four fasted spot UIC.	Vegan (53) (20,33)	56.0 (27.0–586.0) ^2,^*	Mild
		Vegetarian (53) (17,36)	75.0 (1.0–610.0) ^2^	Mild
		Omnivore (100) (37,63)	83.0 (22.0–228.0) ^2^	Mild

M, male; F, female; ^1^, median (25th–75th percentile); ^2^, median (range); ^3^, mean ± SD; * significantly different between dietary group comparison; *p* < 0.05, ** *p* < 0.001; criteria for iodine deficiency disorders (WHO); severe < 20 µg day^−1^, moderate 20–49 µg day^−1^, mild 50–99 µg day^−1^, adequate 100–199 µg day^−1^, excessive risk of adverse health consequences ≥ 300 µg day^−1^.

**Table 4:** Studies investigating iodine among vegans, vegetarians, and omnivores in industrialised countries.

Study, Year	Study Design	Location	Dietary Groups	Sample (*n*) (Male, Female)	Method of Dietary Classification	Average Diet Adherence (Years)
Alles, 2017 [36]	Cross-Sectional	France	Vegan	789	Assessed by investigators pre-study	NA
			Vegetarian	2370		
			Omnivore	90,664		
Draper, 1993 [37]	Cross-sectional	London, UK	Vegan	38 (18,20)	Self-reported	1.0
			Lacto-Vegetarian	52 (16,36)		2.0
			Demi-vegetarian	37 (13,24)		5.0–9.0
Elorinne, 2016 [48]	Matched pairs by age and sex	Kuopio, Finland	Vegan	22 (16,6)	Self-reported	8.6
			Omnivore	19 (11,8)		NA
Henjum, 2018 [38]	Cross-Sectional	Norway, eastern and western geographical regions	Vegan	27	Self-reported	NA
			Vegetarian	9		
			Omnivore	367		
Kristensen, 2015 [49]	Matched pairs by age	Denmark	Vegan	75 (36,39)	Self-reported	≤1.0
			Omnivore	1627 (716, 911)		NA
Krajcovicová-Kudlácková, 2003 [39]	Cross-sectional	Slovakia	Vegan	15 (6,9)	Self-reported	9.7
			Vegetarian	31 (12,19)		9.0
			Omnivore (Mixed Diet)	35 (15,20)		NA
Leung, 2011 [40]	Cross-sectional	Boston, Massachusetts	Vegan	63	Self-reported	11.3 ± 11.7 ^1^
			Vegetarian	78		5.6 ± 5.7 ^1^
Lightowler, 1998 [41]	Cross-sectional	London and surrounding counties, UK	Vegan	30 (11,19)	Self-reported	M, 10.0, F, 9.2
Lightowler, 2002 [42]	Cross-sectional	London and the south-east of England, UK	Vegan	26 (11, 15)	Self-reported	M, 9.9, F, 11.7
Nebl, 2019 [43]	Cross-sectional	Hanover, Germany	Vegan	27 (11,16)	Assessed by investigators pre-study	>2.0
			Vegetarian	25 (10, 15)		>2.0
			Omnivore	27 (10,17)		>3.0
Rauma, 1994 [50]	Matched pairs	Kuopio, Finland.	Vegan (Living Food Diet)	12	Self-reported	6.7 ± 3.8 ^1^
			Omnivore	12		NA
Remer, 1999 [47]	Repeated-measures	Germany	Vegetarian (Lacto)	6 (3,3)	Allocated by investigators	0.0
			Omnivore	6 (3,3)		
			Omnivore (High protein)	6 (3,3)		
Schüpbach, 2017 [44]	Cross-sectional	Lausanne and Zurich, Switzerland	Vegan	53 (20,33)	Self-reported	≤1.0
			Vegetarian	53 (17,36)		≤1.0
			Omnivore	100 (37,63)		≤1.0
Sobiecki, 2016 [45]	Cross-sectional	Oxford, UK	Vegan	803	Assessed by investigators pre-study	≤1.0
			Vegetarian	6673		≤1.0
			Pescatarian	4531		≤1.0
			Omnivore (Meat-eaters)	18,244		≤1.0
Waldmann, 2003 [46]	Cross-sectional	Hanover, Germany	Vegan (Strict)	98	Assessed by investigators pre-study	4.3
			Vegan (Moderate)	56		3.4

NA, not assessed; M, male; F, female; ^1^, mean ± SD.

**Table 5:** Assessment of dietary iodine intake for vegans, vegetarians, and omnivores in industrialised countries.

Study, Year	Assessment of Dietary Iodine	Criteria for Iodine Intake Used in Study	Dietary Group (N) (Male, Female)	Dietary Iodine Intake (µG Day^−1^)	Contribution of Iodised Salt, Seaweed, and Iodine-Containing Supplements	Meeting Criteria (Y/N)
**Allès, 2017** [36]	Three repeated 24 h dietary records.	150 µg day^−1^ RDI for the French population (2001) [52].	Vegan (789) Vegetarian (2370) Omnivore (90,664)	248.3 ± 9.8 (a) ^1^ 222.6 ± 5.7 (a) ^1^ 180.1 ± 1.1 (a) ^1,^**	Seaweed, salt, or supplements not measured.	Y Y Y
**Draper, 1993** [37]	Three-day weighted food diaries. Analysed using UK Ministry of Agriculture, Fisheries and Food data.	DRV of 140 µg day^−1^ Department of Health (1991) [53].	Vegan (38) (18,20)	M, 98.0 ± 42.0 ^2,^** F, 66.0 ± 22.0 ^2,^**	95% used sea salt or seaweed. 30%–40% consumed food supplements containing seaweed 1–2 days a month. 15.6 µg day^−1^ provided by dietary supplements.	N
			Lacto-Vegetarian (52) (16,36)	M, 216.0 ± 73.0 ^2,^** F, 167.0 ± 59.0 ^2,^**	No iodine provided by salt, seaweed or supplements.	Y
			Demi-Vegetarian (35) (13,24)	M, 253.0 ± 164.0 ^2,**^ F, 172.0 ± 91.0 ^2,^**	No iodine provided by salt, seaweed or supplements.	Y
**Kristensen, 2015** [49]	Four-day weighed food diary.	150 µg day^−1^ NNR (2012) [54].	Vegan (70) (33,37)	M, 64.0 (43.0–91.0) ^3,^** F, 65.0 (54.0–86.0) ^3,^**	Salt not measured. Three vegans consumed seaweed. 9.0 µg day^−1^ (M) and 6.0 µg day^−1^ (F) was provided by dietary supplements.	N
			Omnivore (1257) (566,691)	M, 213.0 (180.0–269.0) ^3^ F, 178.0 (146.0–215.0) ^3^	Salt not measured. No iodine provided by seaweed. 107 µg day^−1^ (M) and 78.9 µg day^−1^ (F) was provided by dietary supplements.	Y
**Lightowler, 1998** [41]	Four-day weighed food diary with duplicate portion technique.	140 mg day^−1^ RNI Department of Health (1991) [53].	Vegan (30) (11,19)	M, 138.0 ± 149.0 ^2^ F, 187.0 ± 246.0 ^2^	Salt not measured. Three vegans consumed seaweed, resulting in significantly higher iodine intake (*p* < 0.001) Seaweed consumers were over six times the RNI. Iodine-containing supplements were consumed by five (45%) males and seven females (37%). Providing 54.0 mg day^−1^ on average to the diet.	M, N F, Y
**Lightowler, 2002** [42]	Four -day food diaries with duplicate portion technique. Analysed using CompEat 4 software.	140 mg day^−1^ RNI Department of Health (1991) [53].	Vegan (26) (11,15)	Diet Diary M, 42.0 ± 46.0 ^2^ F, 1448.0 ± 3879.0 ^2^ Duplicate Diary M, 137.0 ± 147.0 ^2^ F, 216.0 ± 386.0 ^2^	Salt not measured. Two vegans consumed seaweed, resulting in iodine intake to exceed the RNI. Dietary supplement intake was recorded but not included to dietary intake.	Diet Diary M, N F, Y Duplicate DiaryM, N F, Y
**Nebl, 2019** [43]	Three-day food diaries analysed by PROD16.4^®^.	200 µg day^−1^ RV German, Austrian and Swiss Nutrition Societies (2019) [55]	Vegan (27) (10,17)	57.7 (48.4, 67.0) ^4,^*	Salt or seaweed not measured. No iodine provided by supplements.	N
			Vegetarian (25) (10,15)	61.6 (49.4, 73.7) ^4,^*		N
			Omnivore (27) (11,16)	88.8 (64.1, 114.0) ^4,^**		N
**Rauma, 1994** [50]	Seven-day food diaries analysed by NUTRICA Finland.	0.1–0.2 mg day^−1^ RDA (120–200 µg day^−1^) Committee on Dietary Allowances, Food and Nutrition Board, National Research Council (1989) [56].	Vegan (Living Food Diet) (9)	29.0 ± 18.0 ^2^	One participant did not use iodised salt. 25% of daily iodine in vegans was provided by seaweed (estimated >8.0 µg day^−1^). Four vegans consumed seaweed, resulting in higher intake.	N
			Omnivore (8)	222.0 ± 93.0 ^2^		Y
**Remer, 1999** [47]	Five-day dietary intervention of pre-selected food items representing each diet. Calculated using food tables.	NA	Vegetarian (Ovo-Vegetarian)(6) Omnivore (6)	15.6 ± 21.0 ^2^ 35.2 ± 15.0 ^2^	No iodized salt, seaweed or supplements were permitted during the study. All drinks including water were low in iodine and other minerals.	N N
			Omnivore (High Protein) (6)	44.5 ± 16.5 ^2^		N
**Waldmann, 2003** [46]	Pre-study questionnaire identifying regularly consumed foods. Two estimated nine-day FFQs using 7 days of records.	200 mg day^−1^ RI, German Society of Nutrition (2000) [57]	Vegan (Strict) (98) (48,50) Vegan (Moderate) (56) (19,37)	M, 87.7 ± 30.6 ^2^ F, 82.1 ± 34.4 ^2^ M, 93.7 ± 27.8 ^2^ F, 78.1 ± 25.6 ^2^	Salt not measured. Seaweed intake not measured. 46% of participants used some form of nutritional supplement. Iodine-specific supplements were not recorded.	N N
**Sobiecki, 2016** [45]	112-item semi-quantitative FFQ. Analysed based on UK Ministry of Agriculture, Fisheries and Food data.	150 µg day^−1^ RDA, dietary reference intakes for iodine (2001) [58]	Vegan (803) (269,534)	M, 55.5 ± 40.0 ^2^ F, 54.1 ± 40.0 ^2^ Total, 58.5 (a) ^2^	Salt not measured. Two participants who consumed seaweed had values close to the maximum tolerable daily intake for iodine. Supplement intakes recorded did not specify iodine content.	M, N F, Y (a), N
			Vegetarian (6673) (1516,5157)	M, 141.0 ± 77.4 ^2^ F, 146.1 ± 78.8 ^2^Total, 148.1 (a) ^2^		M, N F, N (a), N
			Pescatarian (4431) (782,3749)	M, 197.4 ± 84.7 ^2^ F, 194.8 ± 85.9 ^2^ Total, 196.8 (a) ^2^		Y (a), Y
			Omnivore (Meat-Eaters) (18,244) (3798,14446)	M, 214.3 ± 85.6 ^2^ F, 213.8 ± 85.2 ^2^ Total, 212.2 (a) ^2^		Y (a), Y

Abbreviations; RDI, Recommended Daily Intake; DRV, Daily Recommended Value; NNR, Nordic Nutrition Recommendations; RNI, Recommended Nutrient Intake; RV, Recommended Value; RDA, Recommended Daily Allowance; RI, Recommended Intake; (a), adjusted by age and sex; ^1^, mean ± SEM; ^2^ mean ± SD; ^3^, median (25th–75th percentile); ^4^, mean (95% CI); * significant difference with other dietary groups; *p* < 0.005 ** significant difference with other dietary groups; *p* < 0.001.

**Table 6:** Summary of salt fortification programs present in included studies [59].

Country	Year	Iodate and/or Iodide	Iodine Amount (ppm)	State of Legislation
Boston (U.S.)	1920	Iodide	43	Mandatory
Denmark	1999	Iodide	13	Mandatory
France	1997	Iodide	10–15	Voluntary
Finland	1963	Iodide	25	Voluntary
Germany	1981	Iodate	15–20	Voluntary
Norway	NA	Iodide	5	Voluntary
Slovakia	1966	Iodide	25 ± 10	Mandatory
Switzerland	1922	Both	20–30	Voluntary
UK	NA	Iodide	10–22	Voluntary

## Supplementary Materials

The following are available online at https://www.mdpi.com/2072-6643/12/6/1606/s1. Figure S1: Funnel plot showing iodide status for individual groups according to number of individuals in each group. For each study, data for urinary iodide concentration (µg L^−1^) was plotted for omnivores, vegetarians and vegans where provided, against the number of participants in each group. Each data point represents a specific group rather than a specific study. Figure S2 Funnel plot showing iodide consumption for individual groups according to number of individuals in each group. For each study, data for dietary iodide intake (µg Day^−1^) was plotted for omnivores, vegetarians and vegans where provided, against the Log_10_ of the number of participants in each group. Each data point represents a specific group rather than a specific study. Table S1: Search terms used in the study, Table S2: Quality assessment questions, Table S3: Definitions and characteristics of dietary groups studied in included studies, Table S4: National data corresponding to location of included studies, Table S5:Common methods for assessing Dietary Iodine Intake in Population Studies.
