# Supplementary material for: Vegans, Vegetarians, and Omnivores: How Does Dietary Choice Influence Iodine Intake? A Systematic Review
Source: Nutrients. 2020 May 29;12(6):1606. doi: 10.3390/nu12061606 (PMC7352501; doi:10.3390/nu12061606)
Supplement: Supplementary file 1 [file nutrients-12-01606-s001.pdf]

Supplementary Table 1. Search terms used in the study

1	exp Iodine/
2	Iodine*.mp
3	Iodine* Status*
4	Iodine* Intake*
5	Exp Deficiency
6	Exp Hypothyroidism
7	Vegetarian*.mp.
8	Exp Diet, Vegetarian/
9	Vegan*.mp.
10	Exp Diet, Vegan/
11	Fruitarian*.mp.
12	Flexatarian*.mp.
13	Pescatarian*.mp
14	Herbivore*.mp.
15	Plant?Based*.mp.
16	1 OR 2 OR 3 OR 4 OR 5 OR 6
17	7 OR 8 OR 9 OR 10 OR 11 OR 12 OR 13 OR 14 OR 15
18	16 AND 17

Limit all to ‘Humans’, ‘Adult (18 years)’ and ‘English Language’

Supplementary Table 2. Quality assessment questions.

Question	Observational Cohort and Cross-Sectional	Controlled Intervention	Quality Assessment of Case-Control
1	Was the research question or objective in this paper clearly stated?	Was the study described as randomized, a randomized trial, a randomized clinical trial, or an RCT?	Was the research question or objective in this paper clearly stated and appropriate?
2	Was the study population clearly specified and defined?	Was the method of randomization adequate (i.e., use of randomly generated assignment)?	Was the study population clearly specified and defined?
3	Was the participation rate of eligible persons at least 50%?	Was the treatment allocation concealed (so that assignments could not be predicted)?	Did the authors include a sample size justification?
4	Were all the subjects selected or recruited from the same or similar populations (including the same time period)? Were inclusion and exclusion criteria for being in the study prespecified and applied uniformly to all participants?	Were study participants and providers blinded to treatment group assignment?	Were controls selected or recruited from the same or similar population that gave rise to the cases (including the same timeframe)?
5	Was a sample size justification, power description, or variance and effect estimates provided?	Were the people assessing the outcomes blinded to the participants' group assignments?	Were the definitions, inclusion and exclusion criteria, algorithms or processes used to identify or select cases and controls valid, reliable, and implemented consistently

			across all study participants?
6	For the analyses in this paper, were the exposure(s) of interest measured prior to the outcome(s) being measured?	Were the groups similar at baseline on important characteristics that could affect outcomes (e.g., demographics, risk factors, co-morbid conditions)?	Were the cases clearly defined and differentiated from controls?
7	Was the timeframe sufficient so that one could reasonably expect to see an association between exposure and outcome if it existed?	Was the overall drop-out rate from the study at endpoint 20% or lower of the number allocated to treatment?	If less than 100 percent of eligible cases and/or controls were selected for the study, were the cases and/or controls randomly selected from those eligible?
8	For exposures that can vary in amount or level, did the study examine different levels of the exposure as related to the outcome (e.g., categories of exposure, or exposure measured as continuous variable)?	Was the differential drop-out rate (between treatment groups) at endpoint 15 percentage points or lower?	Was there use of concurrent controls?
9	Were the exposure measures (independent variables) clearly defined, valid, reliable, and implemented consistently across all study participants?	Was there high adherence to the intervention protocols for each treatment group?	Were the investigators able to confirm that the exposure/risk occurred prior to the development of the condition or event that defined a participant as a case?

10	Was the exposure(s) assessed more than once over time?	Were other interventions avoided or similar in the groups (e.g., similar background treatments)?	Were the measures of exposure/risk clearly defined, valid, reliable, and implemented consistently (including the same time period) across all study participants?
11	Were the outcome measures (dependent variables) clearly defined, valid, reliable, and implemented consistently across all study participants?	Were outcomes assessed using valid and reliable measures, implemented consistently across all study participants?	Were the assessors of exposure/risk blinded to the case or control status of participants?
12	12. Were the outcome assessors blinded to the exposure status of participants?	Did the authors report that the sample size was sufficiently large to be able to detect a difference in the main outcome between groups with at least 80% power?	Were key potential confounding variables measured and adjusted statistically in the analyses? If matching was used, did the investigators account for matching during study analysis?
13	Was loss to follow-up after baseline 20% or less?	Were outcomes reported or subgroups analysed prespecified (i.e., identified before analyses were conducted)?	-
14	Were key potential confounding variables measured and adjusted statistically for their impact on the relationship between exposure(s) and outcome(s)?	Were all randomized participants analysed in the group to which they were originally assigned, i.e., did they use an intention-to-treat analysis?	-

Supplementary Table 3. Definitions and characteristics of dietary groups studied in included studies.

Dietary Group	Characteristics
Vegan	Avoids all products of animal origin.
Vegan (Living Food Diet)	Consumption of lactobacillus rich foods, avoids products of animal origin.
Vegan (Strict)	Avoids all products of animal origin.
Vegan (Moderate)	Aims to avoid all products of animal origin with occasional consumption of diary and/or eggs.
Vegetarian	Consumption of dairy and eggs but no meat or fish.
Lacto-Vegetarian	No consumption of meat or eggs.
Pescatarian	Consumption of dairy, eggs and fish but not meat.
Demi-vegetarian	Occasional consumption of products of animal origin.
Omnivore (Meat Eaters)	No dietary restrictions or avoidance.

Supplementary Table 4. National data corresponding to location of included studies.

Country	Population	Year	MUIC* (μg/L)	Adequacy	WHO IDD Criteria (1994)
Boston (U.S.)	National	2008	164	Adequate	Optimal
Denmark	Adults (16-65)	2012	145	Adequate	Optimal
France	National, Adults (18-74)	2006-7	136	Adequate	Optimal
Finland	National, Adults (25-64)	2013	83	Insufficient	Mild
Germany	National, Woman (18-79)	2008-2011	54	Insufficient	Mild
Norway	National, Adults (25-64)	2003-4	75	Insufficient	Mild
Slovakia	National, School Aged Children (6-12)	2002	183	Adequate	Optimal
Switzerland	National, Woman Reproductive Age	2015	86	Insufficient	Mild
UK	National, Woman Reproductive Age (16-49)	2013	177	Adequate	Optimal

Supplementary Table 5. Common methods for assessing Dietary Iodine Intake in Population Studies.

Method	Dietary Record or Food Dairy Diary	Weighed Food Diary	Duplicate Diet	Food Frequency Questionnaire (FFQ)	Semi quantitative FFQ	24-h Food Recalls
**Description**	Food and beverages are recorded at consumption. Iodine intake is determined by regional composition data considering seasonal variation.	Food and beverages are weighed and logged into a food record at consumption by the individual or investigator.	Duplicate portions of all food and beverages consumed during a set period are collected and chemically analysed.	A limited checklist of foods and beverages associated with frequency response. Individuals report item consumption over a specific duration.	A checklist of foods and beverages associated with frequency response using standardized portions.	A structured interview capturing detailed information about food and beverages consumed by an individual over the past 24 hours.

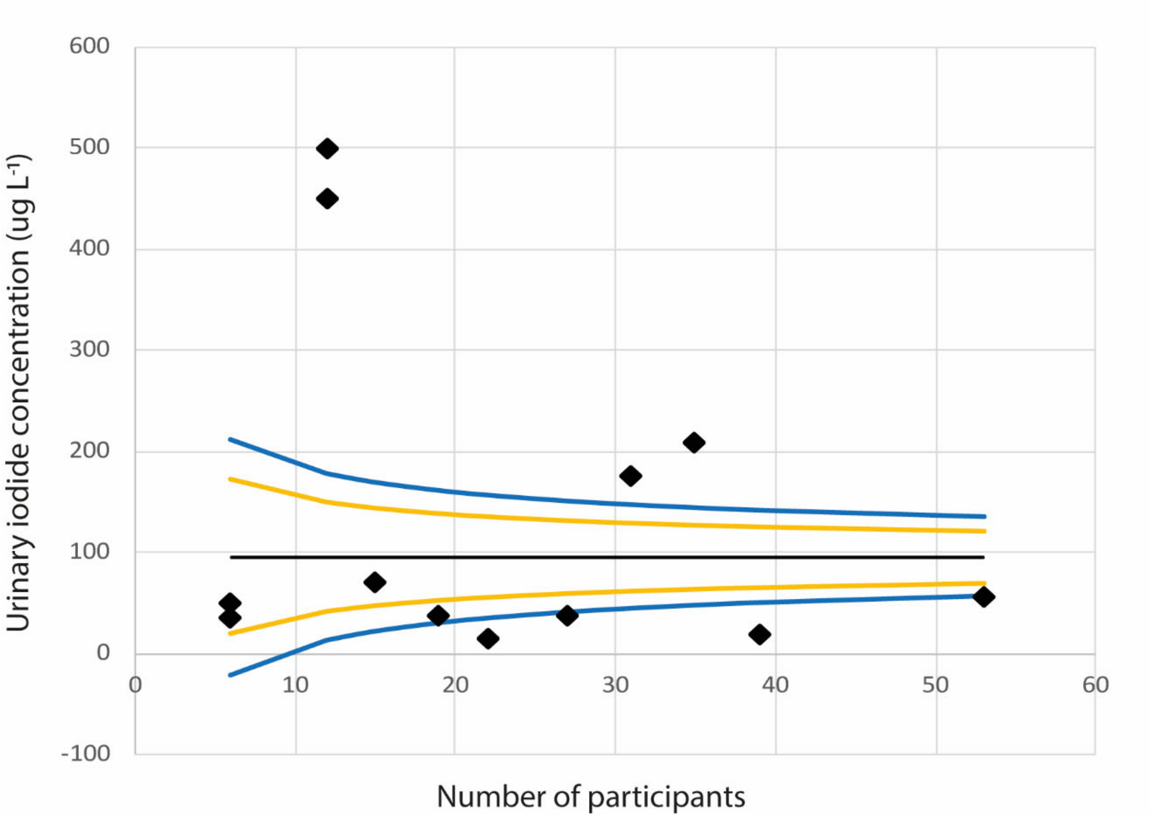

Supplementary Figure 1. Funnel plot showing iodide status for individual groups according to number of individuals in each group. For each study, data for urinary iodide concentration (μg L^{-1}) was plotted for omnivores, vegetarians and vegans where provided, against the number of participants in each group. Each data point represents a specific group rather than a specific study.

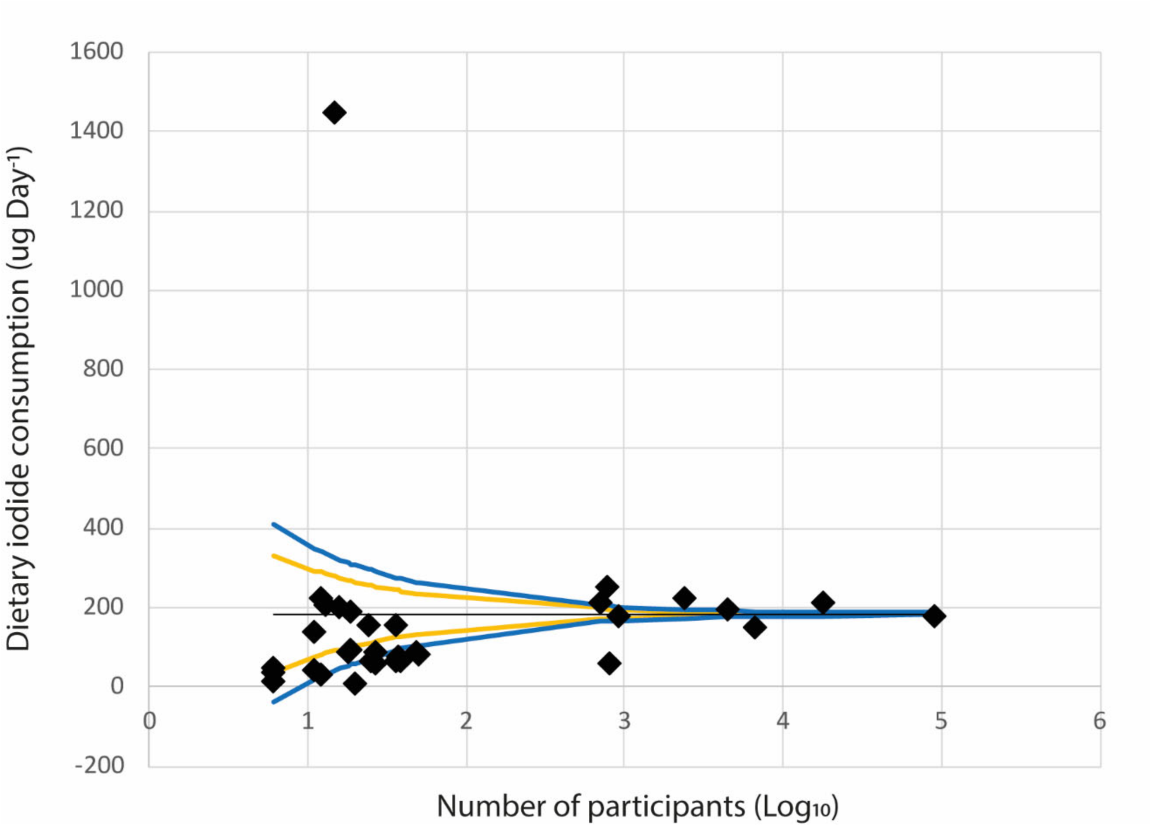

Supplementary Figure 2. Funnel plot showing iodide consumption for individual groups according to number of individuals in each group. For each study, data for dietary iodide intake (μg Day^{-1}) was plotted for omnivores, vegetarians and vegans where provided, against the Log_{10} of the number of participants in each group. Each data point represents a specific group rather than a specific study.
